# Address of Professor Julien S. Sherman, Delivered before the Alumni Association of Chicago Medical College

**Published:** 1870-04

**Authors:** 


### ADDRESS OF PROFESSOR JULIEN S. SHERMAN, DELIVERED BEFORE THE ALUMNI ASSOCIATION OF CHICAGO MEDICAL COLLEGE.

---

ALUMNI OF CHICAO MEDICAL COLLEGE:

*Gentlemen:* — This day closes another year in the history of this Association. We meet for the purpose of renewing the social acquaintance that we formed during our student life. To see the faces and hear the voices of those who, from the noisy medical students, have been transformed into reliable and useful physicians; to take each other by the hand and feel we meet as friends, bound by a common bond of interest in our *Alma Mater;* and as workers in our profession we should come together in perfect harmony. The unmanly tongue that resorts to slander and falsehood should be quiet when it enters here. The science of medicine needs in its ranks those who are searchers of truth. There should be no trickery of Pathys here. The term physician is too noble a word to be disgraced by any such modification. It comprehends all that is known of the laws of health and disease.

He who skilfully uses this knowledge, and confers the greatest benefit to his patient, is the true physician. It becomes the duty of us all to strive that we may merit this title, in its proper signification. To do so we must first be diligent; success will seldom follow those who do not help themselves. Cultivate a friendly and courteous disposition towards your brother practitioners. Do not let the evil spirit of jealousy find a place in your heart. You can never build a monument to yourselves upon the ruins of others. Treat all your patients with kindness and truthfulness; they ask for your skill and expect your sympathy. Communicate to the profession the result of your experience and investigation, so that each of you, during his life may add something to its general stock. This is a duty you owe to the science for its generosity in freely opening its store-house to you. You have the accumulation of ages at your disposal, and you should add something to it, that those who come after you may be benefited by your labors.

Do not be discouraged by reverses. The path to success is not all smooth; there are many rough and rugged places to pass over; but let them arm you with greater zeal, and at last, with such effort you will conquer, and make your lives a pleasure to yourselves and useful to all around you.

Steadily has our *Alma Mater* advanced in her prosperity. The ground is already broken for the construction of an elegant building in connection with the Mercy Hospital, where her students may derive all the benefits which occur from the union of the lecture-room and the bedside of the sick. Her doors are open to all who wish to learn, and the opportunities for obtaining a medical education are extended to females desiring to pursue the study. She numbers at present such an one among her *ad eundem* graduates, whose medical ability is already established. Should others who follow her acquit themselves as she, our *Alma Mater* will never have cause to regret the step she has taken. Time will bring to her still greater progress, and our next annual meeting will be in what me may not inaptly call a new home. By extending her course and increasing her facilities for instructing, she strives to elevate the standard of medical education, and give to the public, from her graduates, those who are gentlemen, scholars, and physicians. With such an object, her progress must ever be upward and onward; and we, her children, will always look back to her with gratitude and pride.

She will have a lively interest in you, and expect from each the deportment becoming the profession you follow.

The year has passed without bringing upon me the painful necessity of announcing the death of any of the members of this association. A Providence in whose hands all things are just has watched and preserved. May such be our record for many years to come.

To those who meet us to-day for the first time, in behalf of the Society, I extend to you an earnest welcome. As me bers of this body, we wish you to feel that you are identified with all its interests, and that you are under obligations to labor for its welfare. For its social standing, it must look to the conduct of its members; and each of you, as you come before the public, will exert your influence for good or evil. It is the little rivulet that forms the mighty river, and the single strokes that fell the oak.

Individuals form society, and each has a personal responsibility in its character. Either in person or by letter represent yourselves at the following meetings. I hope to see the day when these reunions will be attended by members, from every portion of the North-west, and that we will look forward to them as bright and happy spots in our lives. They will serve to keep up our interest in each other and as a recreation from the toils of professional life. As we develop, we can exert more and more our influence on the profession for good. And in helping onward this science, you are conferring a public benefit, which cannot be estimated in the weight of gold. Ask the parent how many dollars and cents will recompense for the loss of his child. What is the value of a useful limb! and what will you take for your vision? These, gentlemen, are materials which are not found in market quotations.

The safety of life will be placed in your hands, as you pass through the world. How necessary it is that you keep yourselves well prepared to meet all emergencies? Every class look to your profession for help—the rich and the poor share its blessings, the strong and the weak are obliged to seek its aid. It carries its influence to the palace hall and to the pauper's hut. On it rests the hopes of loving ones, when disease and suffering enter their homes. You will be called to the mother's side when her babe is born, and you will watch the failing pulse of old age, as the heart that has worked so long grows weary and weak, and asks for rest.

Your fame will not equal that of the warrior or statesman, nor will your graves be crowned with stately monuments. Your deeds will be more hidden from the public; but when you are gone there will be memories of you that will last when marble and brass have crumbled to dust.

Gentlemen, we enter the fourth year of our existence with prosperous omens. Each meeting has increased not only the number in attendance, but the interest shown in the Association. Let each one lend his aid with a will, so that when we meet another year, we may be better and wiser men in the profession of our choice.

